# Portable acuity screening for any school: validation of patched HOTV with amblyopic patients and Bangerter normals

**DOI:** 10.1186/s12886-017-0624-y

**Published:** 2017-12-04

**Authors:** Maya Tsao Wu, M. Diane Armitage, Claire Trujillo, Anna Trujillo, Laura E. Arnold, Lauren Tsao Wu, Robert W. Arnold

**Affiliations:** Alaska Blind Child Discovery, Alaska Children’s EYE & Strabismus, 3500 Latouche #280, Anchorage, Alaska 99508 USA

## Abstract

**Background:**

We needed to validate and calibrate our portable acuity screening tools so amblyopia could be detected quickly and effectively at school entry.

**Methods:**

Spiral-bound flip cards and download pdf surround HOTV acuity test box with critical lines were combined with a matching card. Amblyopic patients performed critical line, then threshold acuity which was then compared to patched E-ETDRS acuity. 5 normal subjects wore Bangerter foil goggles to simulate blur for comparative validation.

**Results:**

The 31 treated amblyopic eyes showed: logMAR HOTV = 0.97(logMAR E-ETDRS)-0.04 r2 = 0.88. All but two (6%) fell less than 2 lines difference. The five showed logMAR HOTV = 1.09 ((logMAR E-ETDRS) + .15 r2 = 0.63. The critical-line, test box was 98% efficient at screening within one line of 20/40.

**Conclusion:**

These tools reliably detected acuity in treated amblyopic patients and Bangerter blurred normal subjects.

These free and affordable tools provide sensitive screening for amblyopia in children from public, private and home schools. Changing “pass” criteria to 4 out of 5 would improve sensitivity with somewhat slower testing for all students.

## Background

Visual acuity screening in early elementary school age is an important safety net for children with undiagnosed or persistent amblyopia that has not yet been detected by a pediatrician in the medical home. In public schools, this task is usually performed by the school nurse. Students in private schools and in home schools often lack the deliberate, scheduled vision screening of a nurse. Acuity screening is an important task and there have been several attempts to simplify this important task including making it available in locations where a computer monitor is available [1].

The Alaska Blind Child Discovery (ABCD) has attempted to eliminate amblyopia by conducting statewide photoscreening of younger children [2] and by enhancing patched monocular acuity screening for older children [3]. Two devices were developed; a quick, portable acuity chart and rolls of patches designed to facilitate affordable, clean, monocular testing. Until now, the devices lacked calibration against the industry standard visual acuity (E-ETDRS). PEDIG has done this with their EVA acuity program so we empoyed their calibration scheme in this study [4].

We validated an inexpensive Flip-card, and a free- download acuity screening tool on known amblyopic patients. Industry standard for young children employs surround HOTV optotypes [5] whereas the standard for older children is the Electronic- Early Treatment of Diabetic Retinopathy Study (E-ETDRS) protocol [6]. Since HOTV optotypes are not copyright protected, they can be available for free internet download. The exhaustive validation of a screening device and calibration of an acuity test often takes a large number of patients with a wide range of disease severity. A Bangerter foil is a vinyl film with irregular surface creating graded amounts of blur (http://www.bernell.com/product/3118/).We used Bangerter foils on normal subjects to do a comparative validation and calibration confirmation study.

## Methods

This cross-sectional study of patients with amblyopia, and then normals is conducted by the Alaska Blind Child Discovery with Institutional Review Board through Providence Hospital. It is compliant with HIPAA and the Declaration of Helsinki. Parents provided informed consent. Three acuity tools are compared: 1) a plastic flip card set, 2) a free computer download Adobe Acrobat file are compared with 3) the E-ETDRS protocol delivered on a calibrated M&S acuity system (Niles, Illinois). The primary outcomes were calibration of the HOTV flip card against the E-ETDRS standard and validation as a screening tool for amblyopia. The secondary outcome was how the device performed with normal subjects with Bangerter foil blur.

### Flip- cards

**Fig. 1:**
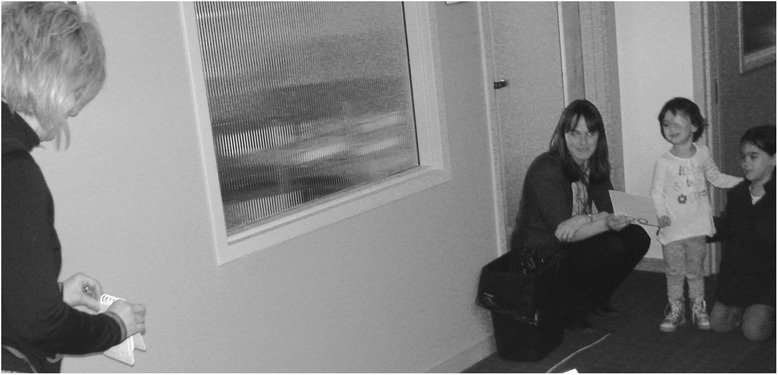
Alaskan school nurse using the ABCD surround, HOTV flip card critical line with matching card on a child wearing a “No Peeking Eye Patch” to assure sensitive, monocular visual acuity screening. Ten foot (3 meter) testing distance assured by accompanying beaded cord- on the floor

A flip card surround HOTV set was modified to range from 20/100 down to 20/16 presented at 10-ft with age-based critical lines 20/32 and 20/40 easily identified by 4 larger cards. In addition, a near card child-calibrated for presentation at 10 in.—instead of 14 in. for adults—is added. An instruction card describes a slower threshold screening protocol and a faster age-based critical line protocol. This acuity flip card set also has a matching card with the four surround HOTV optotypes at 20/80 size and a cord for measuring 10 in. and 10 ft (Fig. 1). This set is manufactured by Precision Vision, LaSalle, Illinois. The acuity protocol used was to present a critical line with 4 random optotypes seeking 3 of 4 for pass. If not passed, then move up to the 20/100 presentations. If passed, then move to smaller (20/80) until threshold— the smallest line with at least 3 of 4 correct. If 20/100 not passed, then move to half distance (5 ft) and approximate 20/200.

### Downloaded pdf acuity

**Fig. 2:**
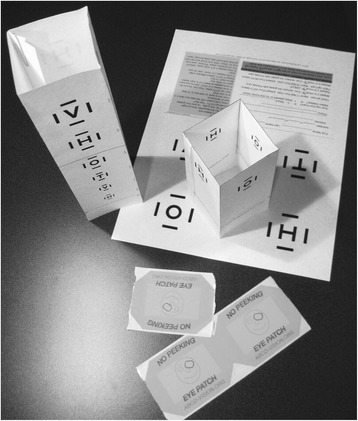
Free, downloadable folded surround HOTV test boxes and matching card with instructions

Kurt Simons, PhD designed an HOTV “top” acuity tester that allows a screener to spin the top to present optoptyes in random fashion. ABCD borrowed Dr. Simons’ simple and elegant idea with his permission. Instead of using the original design, surround, HOTV critical line optotpyes subtending 20/32 and 20/40 at ten feet were printed on an Adobe Acrobat file that can be folded into a test “box” that can be compared to a second pdf file that includes 20/80 sized match card surround HOTV and a set of instructions (Fig. 2). The protocol familiarizes the patient unpatched at 10-in. encouraging pointing to the matching card. Then the screener moves to ten feet from the unpatched child seeking sucessful match. Finally, the non-tested eye is patched and 4 random critical line optotypes are presented with pass if at least 3 of 4 are correct. Both pdf files have been loaded on the www.ABCD-Vision.org website for free download. There is also a link to a video demonstrating use: http://vimeo.com/robertarnold/hotvacuitytestkit .

### Patients

Patients with treated amblyopia in one or both eyes, and already familiar with acuity testing had parental consent and then had the sound eye patched. Amblyopia was defined as best corrected visual acuity of 20/40 or less on E-ETDRS. Each used best spectacle correction and was screened at critical line 20/40 HOTV, then threshold surround HOTV was determined and then the E-ETDRS protocol was performed.

### Bangerter-blurred Normals

Five normal teen female co-authors constructed blur goggles using color-coded swim goggles with a known Bangerter foil (Richmond Products, Albuquerque NM: 0.3, 0.4, 0.6, 0.8 and 1.0) attached to the front of one lens. This was done to provide a range of blurred vision overlapping the screening critical line of 20/40 for Kindergarten entry and younger—or critical line 20/30 (logMAR 20/32) for students older than Kindergarten. The goggles afforded a way to discourage peeking around the Bangerter foil. Testing was performed without routine spectacles. The non-tested eye was occluded with an ABCD patch. Each subject then assessed “pass” (at least three of four correct) or “fail” (less than three of four correct) with no goggles using the ABCD 20/40 folded paper test box, and then sought threshold acuity—smallest line with at least three of four HOTV optotypes correct for each of the Bangerter goggles and with a + 1.0 trial lens for a total of 7 screening conditions each. The gold standard was later tested under each Bangerter-induced blur condition with the E-ETDRS on a subsequent day less than a month later.

## Results

**Fig. 3:**
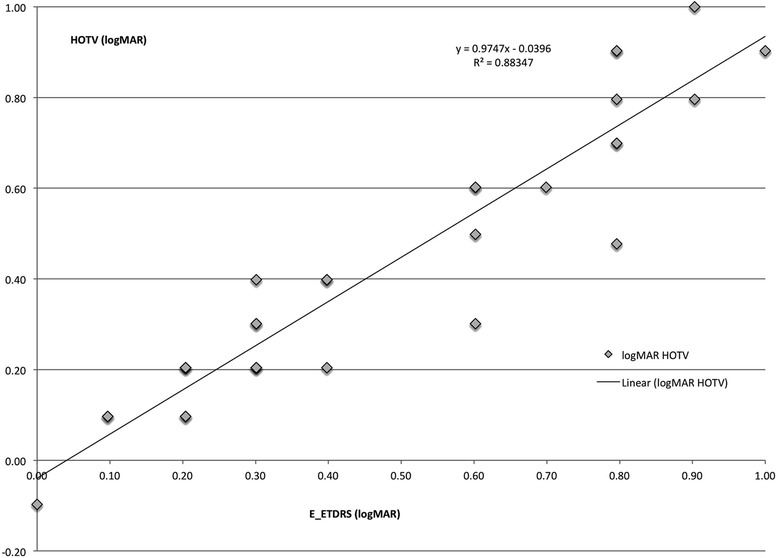
Surround HOTV logMAR acuity versus E-ETDRS logMAR acuity in 31 amblyopic patients

For the 31 amblyopic patients—aged 10.7 ± 3 years, range 6–19 years, a regression between their HOTV threshold logMAR acuity and the E-ETDRS in their worse amblyopic eye is given in Fig. 3. The etiology for these treated, residual amblyopic patients was refractive (anisometropia) in 15, strabismic in 4 and mixed mechanism in 12. Eleven were right eyes. Twelve were girls. The tested eyes had mean ± SD sphere of +1.88 ± 3.4 D with cylinder of +1.38 ± 1.57 D. The spherical equivalent of the tested amblyopic eyes was +3.42 ± 2.51 D. Just two of the 8 patients with astigmatism greater than 2 diopters had an oblique axis (> 10° from axial). Linear regression of HOTV versus E-ETDRS showed good correlation (r [2] = 0.88) with HOTV = 0.97 (E-ETDRS) -0.04 (Fig. 3).

**Fig. 4:**
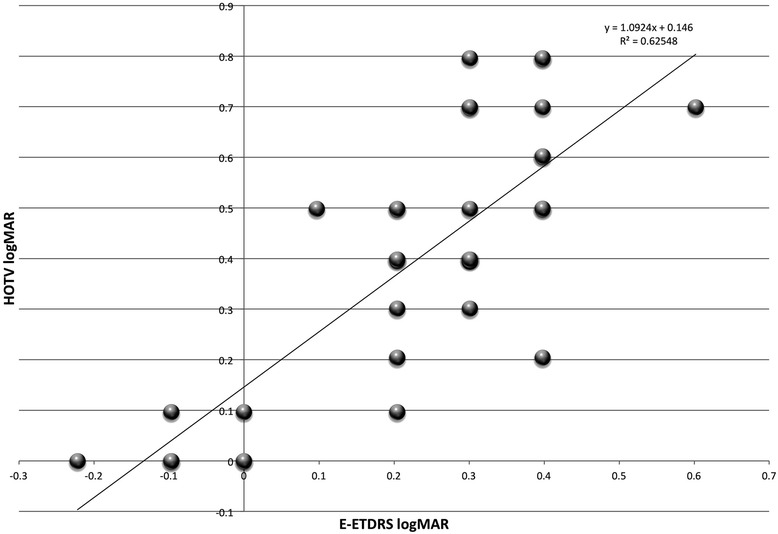
Surround HOTV logMAR acuity versus E-ETDRS logMAR acuity in normal subjects with blur induced by Bangerter foil swim goggles

Five normal teen females provided critical line surround HOTV, then threshold HOTV with five different Bangerter blurred goggles, then with a + 1 trial lens and additionally in their natural state (“none”). The normal subjects all corrected to better than 20/20. Visual acuity in each Bangerter blur condition, the +1 trial and in natural state was repeated on a subsequent day within 1 month with E-ETDRS. A regression of logMAR acuity with critical line identified is shown in Fig. 4.

For the 31 amblyopia patients, mean ± S.D. HOTV acuity was 0.43 ± 0.30 logMAR compared to E-ETDRS of 0.48 ± 0.29 logMAR. For the Bangerter blurred girls, the mean ± S.D. logMAR acuity for HOTV was 0.40 ± 0.25 and for E-ETDRS 0.23 ± 0.18. The difference between E-ETDRS and HOTV for the 31 amblyopic patients was 0.05 (95% C.I. -0.11, 0.25). The difference between E-ETDRS and HOTV for the Bangerter blurred normal subjects was 0.17 (95% C.I -0.03, 0.40).

**Fig. 5:**
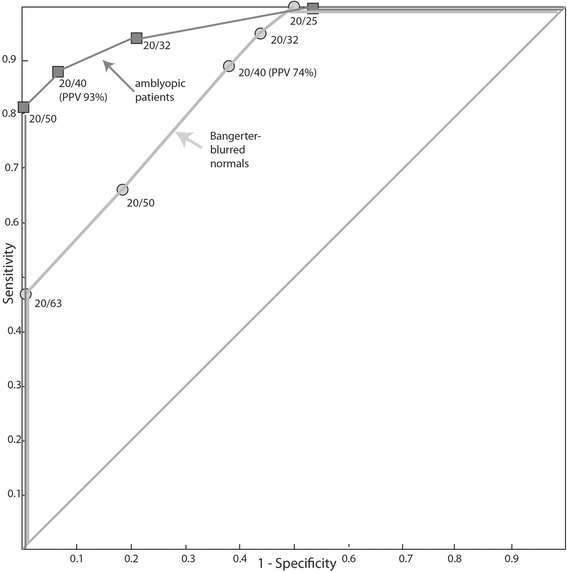
Receiver-operator characteristic (ROC) curve for surround HOTV flip card and test box screening in amblyopic patients and in Bangerter foil blurred normal subjects. Amblyopia is defined as E-ETDRS visual acuity of 20/40 or worse. Points on the curve correspond to passing that given level of HOTV visual acuity with at least 3 of 4 correct

A critical line screening (the pdf download) correctly sorted all but one (94%) of the amblyopic patients within 1 logMAR lines of 20/40 E-ETDRS. This one case was 20/80 E-ETDRS passing 20/40 screening HOTV. Critical line screening correctly (within one logMAR line) identified E-ETDRS acuity in the Bangerter-blurred normals in 34 of 35 (97%) of cases. The positive predictive value of the 20/40 HOTV screening was 93% for the amblyopic patients and 74% for Bangerter blurred normal subjects. The ROC curve for the amblyopic patients and the Bangerter blurred normals is given in Fig. 5.

Using the HOTV and E-ETDRS scales, the Bangerter foil goggles produced a degradation in visual acuity shown in Table 1. Demographics of the amblyopic children are given in Table 2.

**Table 1:** Degree of acuity degradation due to different Bangerter filters placed on color-coded swim goggles

	From baseline	logMAR
Blur goggles	HOTV	E-ETDRS
Bangerter 0.3	0.46	0.56
Bangerter 0.4	0.44	0.48
Bangerter 0.6	0.34	0.50
Bangerter 0.8	0.46	0.68
Bangerter 1.0	0.32	0.28

**Table 2:** Demographics of 31 amblyopia patients showing gender, age, refraction in the amblyopic eye (sphere, plus cylinder and axis), pass or fail the 20/40 critical line, Visual acuity on flip card surround HOTV and on patched E-ETDRS screening. Amblyopic Patient Demographics

Amblyopic Patients									
GENDER	age	eye	type	Sphere	Cyl +	Axis	20/40	logMar ETDRS	logMAR HOTV
F	5.7	L	mix	3.75	0.75	100	pass	0.00	−0.10
M	7.4	L	strab	2	0.75	75	pass	0.10	0.10
M	12.1	L	mix	−1.25	2.25	83	pass	0.10	0.10
M	9.6	L	mix	1.75	0	0	pass	0.20	0.10
M	10.3	l	ref	1.25	4	87	pass	0.20	0.10
F	9.7	R	mix	2.5	2.25	73	pass	0.20	0.20
M	10.7	r	mix	3	.25	15	pass	0.20	0.20
M	8.1	r	mix	1.5	.25	13	pass	0.20	0.20
F	6.6	R	ref	1.5	3.5	92	pass	0.30	0.20
F	13.3	R	mix	4.5	0.5	89	pass	0.30	0.20
M	15.7	l	mix	5.25	.75	119	pass	0.30	0.20
M	10.2	R	ref	−3.5	5	92	pass	0.40	0.20
F	19.9	L	strab	1.25	0.5	125	pass	0.30	0.30
M	17.5	R	mix	6.5	0	0	pass	0.30	0.30
M	7.8	R	mix	3.75	0	0	pass	0.60	0.30
M	10.4	r	mix	2.5	1.5	69	fail	0.30	0.40
M	12.5	l	ref	5	3	108	fail	0.40	0.40
F	9.0	l	strab	4.25	0.25	165	fail	0.40	0.40
M	8.4	L	ref	2.75	0.5	100	fail	0.80	0.48
M	13.9	l	ref	7	1	88	fail	0.60	0.50
M	11.2	l	ref	2.75	2	77	fail	0.60	0.60
F	9.8	l	ref	3.75	1	82	fail	0.60	0.60
M	9.2	l	ref	2	5	96	fail	0.70	0.60
F	7.1	R	ref	0	2.5	96	fail	0.80	0.70
F	14.0	l	strab	5.5	1	115	fail	0.80	0.70
M	11.0	L	ref	6.5	0.5	120	fail	0.80	0.80
M	14.0	R	ref	−7	4	85	fail	0.90	0.80
M	7.4	R	mix	4	0.5	95	fail	0.80	0.90
F	9.2	l	ref	2.25	1	136	fail	0.80	0.90
M	12.6	L	ref	3	0	0	fail	1.00	0.90
F	9.0	r	ref	4.75	2	86	fail	0.90	1.00
mean	10.8			1.88	1.38			0.48	0.43
STDEV	3.2			3.40	1.57			0.29	0.30

## Discussion

This paper addresses two questions: 1) are the surround HOTV flip card and the paper critical line “box” valid for school screening? and 2) can blur by Bangerter filter enhance acuity screen calibration and validation?

The American Academy of Pediatrics (AAP) publishes an evidence-based, age- approriate series of vision screening techniques aimed at detecting amblyopia early when therapy is most effective. Instrument-based screening (photoscreening) can accurately detect amblyopia risk factors in toddlers, but it is preferrable for the instrument referral criteria to be deliberately set for high specificity (catch the most severe cases but not over-refer mild cases) [7]. In order for early specific screening to work [8], more sensitive methods for amblyopia detection must be applied in older children their first school years. A sensitive test makes sure no child with substantial amblyopia can inadvertantly pass the screening. School nurses therefore need an acuity screening tool that will not miss a case of amblyopia worse than 20/40. AAP recommends patching the non-tested eye so no child can peek.

The school nurse is hoping to quickly find children with amblyopia—ideally on school entry—so the visiual disorder will not result in permanent, lifetime vision impairment. School nurses should also identify those children with acuity consistent with good school performance (ie better than 20/30). It is less critical for the school nurse to differentiate between acuity of 20/80, 20/100 and 20/150—under each circumstance that student would need referral. In both the amblyopic patients and the Bangerter-blurred teens, a strong correlation was found for acuity in the better ranges. As a result, the ABCD surround HOTV flip chart—employing the critical line or threshold protocols—and the free downloaded critical-line folded “box” should serve their purpose well. Parents of home-schooled children can, and should, screen their students if this has not been done in a recent pediatrician, well-child examination. For private schools lacking a school nurse, parents, staff, or even upper classmen could perform screening for the early elementary grades. We found the critical line screening to take less than 90 s while threshold patched surround HOTV took 3–4 min. E-ETDRS took 4–7 min in the teens and the older, treated amblyopia patients.

In the amblyopic patients using critical-line screening, two patients passing had worse E-ETDRS acuity (20/50 and 20/80). One patient who failed 20/40 critical line actually had 20/40 E-ETDRS acuity. The advantage of critical line screening is that it takes much less time than threshold screening. The deficiency of critical line/matching is the possibility of correctly guessing. The chance a patient can guess 3 of four matching HOTV optotypes correctly (13/256) is about one in 20. The sensitivity of our test would increase by requiring 4 of 5 correct at critical line; only about 1 in 39 could pass by guessing. This would lengthen the time of testing somewhat.

For the normal girls, the Bangerter foil goggles provided substantial range of blur. The HOTV acuity mean was a line worse than the E-ETDRS, while for the amblyopic patients, the mean HOTV threshold acuity was almost a logMAR line better. The Bangerter blurred patients performed less consistently for higher levels of blur. It may be that the effect on acuity as a result of treated amblyopia may differ from that of Bangerter blur [9].

The Pediatric Eye Disease Investigator Group (PEDIG) compared a repeat, computer-presented surround HOTV patched protocol (EVA) to E-ETDRS and found similarities to our study [4]. The HOTV slightly over-estimated visual acuity by 0.68 logMAR especially for those with amblyopia and less for eyes better than 0.3 logMAR. We found 0.5 lines with our amblyopic patients and 1.7 lines with Bangerter blurred normals. The E-ETDRS was found reliable in older children [6]. Crowded Kay picture acuity has been correlated with ETDRS [10]. The VIPS study found non-copyrighted HOTV and copyrighted LEA Symbols similar for a large number of young, pre-school Headstart students; however, the critical line for HOTV is one logMAR line larger than for LEA Symbols [11].

Bangerter and convex lenses have been used to compare acuity as early as 1984 [12]. Our Bangerter foils were placed on color-coded swim goggles to assure non-peeking and to enable proper identification of each blur severity. The goggles themselves may have added a non-uniform amount of blur evidenced by the non-linear degree of acuity with Bangerter foil power. Future efforts may employ a better form of no-peek frame for the Bangerter foils. The PEDIG group assessed the degree of acuity degradation due to strong Bangerter filters on the spectacles over the sound eye of patients in a clinical trial [13, 14]. They found that Bangerter 0.2 decreased acuity 5.1 lines while Bangerter 0.3 decreased acuity 4.8 lines. In comparison, we found Bangerter 0.3 on goggles to reduce acuity 5.6 lines. Similar to PEDIG, we did not find a linear relationship between acuity degradation and weaker Bangerter stated power (Table 1) [15]. We also suspect our different swim goggles may independently have had variable effect on acuity—but this does not adversely effect a validation / calibration study in which we seek to degrade normal acuity graded amounts equally for comparison between two threshold and one acuity critical line testing methods.

We did not complete an expensive large, multi-center trial of low pre-screening prevalence preschool children. Weaknesses of our study are the lack of a population-based screening with three acuity charts age-appropriate pre-schoolers with confirmatory exams on each- ther referred and the pass- with sufficient numbers to yield a large number of referrals from which to calibrate each tool, and to determine sensitivity and specificity as a screening tool. We did not check test re-Test reliability. We housed the Bangerter foils in color-coded swim goggles- and these may have variably degraded acuity. We included children older than pre-school age. Multi-center, population –based vision screening validation is prohibitively expensive for many of the manufactureres, and we lacked the millions of federal dollars needed to carry out a more ideal, multi-year study.

We saved time and money. Strengths of our study were comparing methodology with PEDIG acuity test calibration [4], the demonstration of a novel method for attaining a larger number of paired comparisons by variably blurring cooperative normal subjects, and the presentation of a receiver operator characteristic curve comparing our relatively inexpensive clinical trails to calibrate and validate pediatric vision screening devices.

A recent proposed set of guidelines suggest 20/50 screening for children age 3 and 20/40 for children aged 4 and 5 years old [16]; the Flip card can accomplish this though the pre-printed instruction follow existing AAP guidelines.

## Conclusion

These affordable, portable surround HOTV screening methods seemed to perform well on amblyopia patients. These critical line screening methods are inexpensive and can be quickly used by school nurses, private school staff and home school parents with a small chance of missing a child with amblyopia. Using critical line with 4 out of 5 needed to pass instead of 3 of 4 would improve sensitivity. Bangerter blur may be a way to facilitate acuity validation using normal subjects to approximate more profound levels of amblyopia.

## Abbreviations

AAP: American Academy of Pediatrics
ATS: Amblyopia Treatment Studies – controled multicenter amblyopia studies performed by PEDIG
C.I.: Confidence interval
D: Diopter
E-ETDRS: Electronic Early Treatment of Diabetic Retinopathy Study- uniform visual acuity optotype presentation on computer utilizing ten capital letters.
EVA: Electronic visual acuity- paradigm of presentation of surrounded HOTV optotypes developed for the PEDIG ATS
HIPAA: Health Insurance Portability and Accountability Act of 1996
HOTV: Uniform optotype for visual acuity testing with bilaterally symmetric capical letters
LEA: Pediatric optotypes developed and marketed by Lea Hyvarinen, MD.
logMAR Base: 10 logarithm of minimum angle of resolution- a measure of the size of an optotype viewed at a given distance.
Pdf: Portable Document Format (Adobe Acrobat).
PEDIG: Pediatric Eye Disease Investigator Group.
ROC: Receiver operator characteristic- plot of the relationship between sensitivity and specificity at various referral criteria for a screening test.
SD: Standard deviation.
VIPS: Vision in Preschoolers Study.
